# Draft Genome Sequence of the Sattazolin-Producing Strain *Pseudonocardia* sp. C8, Isolated from a Mud Dauber Wasp Nest in Nepal

**DOI:** 10.1128/MRA.00007-21

**Published:** 2021-03-11

**Authors:** Niraj Aryal, Saefuddin Aziz, Prajwal Rajbhandari, Harald Gross

**Affiliations:** a Department of Pharmaceutical Biology, Institute of Pharmaceutical Sciences, University of Tübingen, Tübingen, Germany; b Microbiology Department, Biology Faculty, Jenderal Soedirman University, Purwokerto, Indonesia; c Department of Applied Microbiology and Food Technology, Research Institute for Bioscience and Biotechnology, Kathmandu, Nepal; d German Centre for Infection Research, Partner Site Tübingen, Tübingen, Germany; University of Maryland School of Medicine

## Abstract

Here, we report a 6.2-Mbp draft genome sequence of the bacterium *Pseudonocardia* sp. strain C8, which gave insight into the complete secondary metabolite production capacity of the strain and hinted that the strain possibly represents a new species.

## ANNOUNCEMENT

As part of our ongoing collaborative effort to investigate bacteria from underexplored resources (1), we isolated and characterized another mud dauber wasp nest-associated actinobacterium. In chemical analyses, it became apparent that strain C8 produces the antiviral compound sattazolin (2–4). In order to investigate the taxonomy and the complete biosynthetic capacity for secondary metabolism of C8, as well as to locate the biosynthetic gene cluster (BGC) coding for sattazolin, the sequencing of this strain was initiated.

The isolation of the strain was conducted as published previously (1). For genomic DNA isolation, strain C8 was grown in seed medium (1) for 8 days at 30°C on a rotary shaker (160 rpm). For genomic DNA isolation, a Quick-DNA fungal/bacterial DNA miniprep kit (Zymo Research, Irvine, CA, USA) was used according to the manufacturer’s protocol except that the vortex-mixing step was reduced from 15 min to 5 min and was conducted at maximum speed. The DNA was sheared using a Covaris g-TUBE, and the genomic library was prepared according to the standard PacBio protocol, followed by size selection with the BluePippin size selection system (Sage Science, Inc.). The 10-kb library was sequenced on a PacBio Sequel instrument using one single-molecule real-time (SMRT) cell, resulting in 2,118,991 reads with a median read length of 7,243 bp. No quality filtering was conducted; however, subreads shorter than 50 bp were discarded. The remaining PacBio long reads were assembled using SMRT Link v7.0.1 and HGAP4 (5, 6). All software settings were kept at their defaults except for the HGAP4 genome size estimate parameter, which was set to 7 Mbp, the average size of an actinobacterial genome. Overall, the reads were assembled into a 6,241,701-nucleotide draft genome at 189-fold coverage. The resulting sequence consisted of two contigs (contig 1, 134,573 bp; contig 2, 6,107,128 bp), with a G+C content of 73.60%. The large contig represented the chromosome, while the small one was expected to be a plasmid, particularly since a BLASTn analysis revealed that contig 1 is highly similar (85.3% identity) to the Pseudonocardia dioxanivorans plasmid pPSED01 (GenBank accession number CP002594.1). Gene functional annotation using PGAP v4.12 (7) identified 5,637 coding genes.

An automated genome-based taxonomic analysis of strain C8, employing the Type Strain Genome Server (TYGS) (8), revealed that Pseudonocardia ammonioxydans CGMCC 4.1877^T^ (9) represents the closest related type strain of C8. In pairwise comparisons, independent of the applied Genome BLAST Distance Phylogeny (GBDP) formula, the digital DNA-DNA hybridization (dDDH) values d0, d4, and d6 did not exceed 32.1%. Since these values are well below the species threshold of 70%, strain C8 possibly represents a candidate new *Pseudonocardia* species. This tentative finding was complemented by an analysis of the average nucleotide identity (ANI) using autoMLST (10), which revealed that the C8 genome sequence had 86.7% ANI to Pseudonocardia ammonioxydans CGMCC 4.1877^T^. Since this ANI value is also well below the one for species delineation (94 to 96%), this analysis supported the TYGS results. Further experiments, which are required to determine the species of C8, will be the subject of future investigations.

Automated secondary metabolism analysis using antiSMASH v6.0.0 (11) predicted 16 BGCs. Only 1 BCG matched at the 100% level the known cluster encoding the compatible solute ectoine (12); the remaining 15 BGCs await further validation.

### Data availability.

This whole-genome shotgun project has been deposited in DDBJ/ENA/GenBank under the accession number JACMTR000000000. The raw sequencing data are available from the Sequence Read Archive (SRA) under the accession number SRR12464673.
